# Animal welfare or animal well-being: A review of terminological use in zoo research and practice

**DOI:** 10.1017/awf.2026.10108

**Published:** 2026-09-14

**Authors:** Lucy Stratford, Lindsay Murray, Katie L. Edwards, Lisa Holmes

**Affiliations:** 1 Division of Psychology, University of Chester, Chester, UK; 2 Chester Zoo, Chester, UK

**Keywords:** Animal welfare, terminology, well-being, zoo animals, welfare assessment, welfare definition

## Abstract

Zoo animal welfare research is a rapidly developing field; however, there is considerable variation in how animal welfare is defined, both across disciplines and authors. This complexity is further complicated by the increasing use of related terms, such as well-being, happiness, and wellness, often with little consensus on their distinct meanings. This paper examines how the terms welfare and well-being are used in zoo-based animal research, exploring whether they are treated as interchangeable or fundamentally different terms. While some authors make a deliberate distinction between the two, a substantial overlap in definitions is evident across the literature, so much so that the distinction often lacks a logical or functional basis. To promote conceptual clarity and prevent further fragmentation of the research field, it is recommended that a single term be used consistently. Specifically, the term welfare is recommended, due to its established grounding in science. Furthermore, rather than reducing welfare to a singular definition, it is advised that researchers should focus on understanding and evaluating its key components, allowing for nuanced and evidence-based assessments of the lived experiences of animals housed under human care in zoos.

## Introduction

As Marian Dawkins states, “*Animal welfare science has been, and indeed still is, bewitched by the words it uses*” (Dawkins 2021; p 84). As a field, animal welfare is rapidly developing, with evolving conceptualisations of welfare leading to variations in the components considered most important. Historically, animal welfare was viewed primarily in relation to the animal’s fundamental, physical needs (Fraser 2008). If an animal was healthy and free from physical suffering, they were considered to have good welfare. Further developments saw emphasis placed on the interactions an animal has with its environment, how it copes and adapts to change, and natural species-typical behaviour, with one of the most cited definitions coming from Broom (1986) as “*the animal’s state as regards its attempts to cope with its environment*” (Broom 1986; p 524). Efforts to avoid anthropomorphising (attributing human characteristics to animals) often prevented the inclusion of how an animal feels within welfare discussions. However, over the last several years there has been a clear shift towards including the affective state and subjective experiences of an animal into welfare conceptualisations; for example, during the early 2010s, several publications highlighted the importance of recognising and promoting positive affective states, introducing concepts such as “*a life worth living*” and “*a good life*” (Mellor 2012, 2016; Mellor & Beausoleil 2015).

The Five Domains Model is perhaps one of the most widely known and well-established frameworks for animal welfare. First developed in 1994, the Five Domains Model is comprised of four Functional Domains (Nutrition, Environment, Health and Behavioural Interactions) and the fifth, Mental Domain (Mellor & Reid 1994; Mellor *et al.* 2020). It has been adapted several times since its initial development to reflect advancements in scientific knowledge, with most recently, the fourth domain, Behavioural Interactions expanded to incorporate interactions with other animals, with humans and with the environment (Mellor *et al.* 2020). The fifth, Mental Domain encompasses all feelings and/or sensations that an animal has across their whole life, providing a holistic overview of their lived experience. All five domains contribute towards the animal’s overall welfare state (Mellor & Reid 1994; Mellor *et al.* 2020) and the Five Domains Model of Welfare remains the foundation of all welfare activities within the zoo sector.

For zoos and aquariums (hereafter referred to as zoos), animal welfare is a top priority (Tallo-Parra *et al.* 2023). It is considered that zoos have four pillars of operating practices: entertainment, education, conservation and research, and promoting high standards of animal welfare is central to all four (Harley & Clark 2019; Rose *et al.* 2025). Animals with better welfare are better candidates for conservation breeding programmes, lead to more valid and reliable research, and also lead to a better educational and overall, more positive, visitor experience (Harley & Clark 2019; Rose *et al.* 2025).

Global and regional-level zoo accreditation bodies are in place to help guide and support zoos in practices relating to animal care, conservation and welfare (WAZA 2026). Zoos can become members of the World Association of Zoos and Aquariums (WAZA), the European Association of Zoos and Aquariums (EAZA) or the British & Irish Association of Zoos and Aquariums (BIAZA), and so on, dependent on geographical location, through a comprehensive accreditation process. To be BIAZA-accredited, for example, zoological organisations are required to exceed minimum requirements relating to husbandry and care, and actively contribute to public education, conservation efforts, research excellence and animal welfare (BIAZA 2025).

Across all of these practices, both at a zoo and a broader welfare-science level, the definition of welfare remains a contentious and ongoing discussion, with little consensus reached. Historical origins of the word ‘welfare’ literally attest to the meaning of ‘fare well’ or doing or going well and, as such, this is not negative (Etymonline, Online Etymology Dictionary: https://www.etymonline.com/word/welfare). However, its adoption in the realm of politics and social reform have seen it equated with the distribution of welfare benefits to those potentially stigmatised as therefore being ‘on welfare’ (Etymonline, Online Etymology Dictionary: https://www.etymonline.com/word/welfare). Such subtleties of wording have been found to make a real difference to how participants respond in surveys; far more negative connotations surround the term ‘welfare’ as compared to terms alluding to ‘caring for the poor’ (Smith 1987). Similarly, negative associations are often made about those in receipt of welfare services; for example, children involved with Child Welfare Services are more at risk of negative life events and mental health issues than the general population (Furuhaug *et al.* 2024). As Phillips (2008; p 291) stated, “*Animal welfare is usually considered primarily in terms of the animal’s negative experiences*”.

For these reasons, some authors and organisations have opted to use synonyms such as happiness, wellness or well-being, particularly in any public-facing documents, (e.g. the Association of Zoos and Aquariums’ [AZA] Strategic Framework for the Well-being of Animal 2022). This, however, introduces the issue of synonyms where there remains little clarity as to whether terms mean the same thing, are independent of one another or are extensions of one another, i.e. one an outcome of another.

As it stands, it appears to be author discretion that determines which term is used and its definition. Cultural differences between North America and Europe have been cited as reasons why researchers and institutions have a preference towards using the term well-being or welfare; well-being, referring to the overall state of the animal is often more accepted in the US, whereas welfare, meaning the wider concept of ethical and social issues related to the animal, is adopted in Europe (Clark *et al.* 1997; Lerner 2008).

It is hypothesised here that well-being may have risen to prominence within the field of welfare science, as a means of emphasising mental state, psychological wellness and affective states, at a similar time as the introduction of “*a good life*” (Mellor 2012). This trend is consistent with the adoption of the term ‘well-being’ by accreditation bodies and government welfare frameworks, whereby the term well-being was integrated into formal definitions of welfare during this period (see the Irish Modern Standards of Zoo Practice 2016 and WAZA’s ‘Caring for Wildlife’ Animal Welfare Strategy 2015). In more recent years, however, there appears to be a renewed emphasis on the term ‘welfare’, reflecting a broader conceptualisation of animals’ lived experience that includes positive affective states along a continuum of welfare (WAZA 2020).

Regardless of term used, a solid understanding of the definition of welfare is needed before any welfare processes, such as assessment or monitoring, can begin (Appleby *et al.* 1996; Mellor & Stafford 2001; Appleby & Sandøe 2002; Weary & Robbins 2019). Animal welfare is now widely recognised as an absolute priority and fundamental component of all operations of the modern zoo (Tallo-Parra *et al.* 2023; Rose *et al.* 2025); however, the lack of consensus on how animal welfare is defined can present a challenge to how it is understood and ultimately assessed (Veasey 2022).

This opinion piece summarises the definitions of welfare in a zoological context, comparing components emphasised and discussing regional and temporal changes. Moreover, this opinion piece attempts to demystify the issue of synonyms, presenting a brief overview of where the terms, welfare and well-being, have been presented as independent components or have been explicitly referred to as interchangeable. Clarifying these definitions and terminologies used within the field of animal welfare science is important for ensuring that welfare is consistently interpreted and applied effectively both within and across zoos and at an accreditation level.

## Defining welfare

Zoo accreditation bodies publish supplementary material designed to support member zoos in advancing their animal welfare standards. These include documents such as WAZA’s Animal Welfare Strategy, Advancing Animal Welfare, and BIAZA’s Animal Welfare Policy (BIAZA 2023; WAZA 2025). Zoo accreditation bodies also offer their own working definitions of welfare, both within these documents, and freely accessible on their websites (e.g. EAZA’s Animal Welfare Resources 2026), designed to help direct member zoos and aquariums to areas of priority. These documents have been developed by researchers and professionals in animal welfare, either through internal Working Groups (such as BIAZA’s Animal Welfare Working Group) or, through expert consultation (+Advancing Animal Welfare Strategy involved over 40 academic and zoo professionals working in the field of animal welfare). How welfare is understood, and thus defined, differs across the documents provided by accreditation bodies. The definitions of welfare provided by zoo accreditation bodies are presented in Table 1. Some definitions have been shortened for ease of presentation and are denoted by […].

**Table 1.** Definitions of Welfare by Zoo Accreditation Bodies at a global and regional level, including AZA, BIAZA, EAZA, WAZA and ZAA Table 1. long description.

Accreditation body	Definition
AZA	“*The state of being comfortable, healthy or happy; achieved by AZA facilities giving animals lifelong opportunities to thrive*” (AZA 2022).
BIAZA	“*The state of the animal as perceived by the animal itself with regards to its attempts to cope with its environment*” (BIAZA 2023).
EAZA	“*The physical, behavioural and social well-being of animals through the provision of appropriate conditions for the species involved, including but not necessarily limited to housing, environment, diet, veterinary care and social contact where applicable. The Five Domains Model of Welfare is the primary model accepted by EAZA for assessing welfare*” (EAZA 2024).
WAZA	“*Animal welfare refers to a state that is specific for every individual animal; it is how the animal experiences its own world and life through its association with pleasant experiences specific for that species such as vitality, affection, safety and excitement, or unpleasant experiences such as pain, hunger, fear, boredom, loneliness and frustration* […]” (WAZA 2020).
ZAA	“*Positive Welfare/Good Welfare: The welfare state of an animal that is achieved when positive mental experiences are promoted and at the same time negative mental experiences are minimised. Negative Welfare/Poor Welfare: The welfare state of an animal when there are negative mental experiences occurring on a severe, frequent or ongoing basis*” (ZAA 2026).

*Note:* AZA ([American] Association of Zoos & Aquariums), BIAZA (British & Irish Association of Zoos & Aquariums), EAZA (European Association of Zoos & Aquariums), WAZA (World Association of Zoos & Aquariums), ZAA (Zoo & Aquarium Association [Australasia]).

Overall, definitions of welfare provided by accreditation bodies vary in length and complexity, with some organisations opting for short, high-level, sentence-long definitions (van der Staay *et al.* 2025). Common components across different definitions of welfare include physical health, natural behaviour, affective state, and how the animal experiences life. Concepts emphasised align closely to those captured within the Five Domains Model of welfare (Mellor *et al.* 2020).

The definitions presented in Table 1 also align with legal definitions of welfare. The Animal Welfare Act (2006) defines welfare in terms of animals’ five needs: the need for a suitable environment; a suitable diet; the ability to express natural behaviours; the need to be housed with conspecifics; and to be protected from injury, pain, disease and suffering (Animal Welfare Act 2006). As part of their zoo licensing agreement, zoos are also legally obligated to meet minimum care standards, relating to animal care, enclosure design, and management practices, among others, as outlined in government-level regulations. In Great Britain, this is the Standards of Modern Zoo Practice (SMZPGB 2025) and in Ireland, the Irish Standards of Modern Zoo Practice (2016). The latter defines welfare as: “*the state of an individual animal* […] *how an animal is coping with the conditions in which it lives and how the animal perceives its experiences* […]” (Irish Standards of Modern Zoo Practice 2016; p 3) and the Standards of Modern Zoo Practice Great Britain define welfare using the same ‘Five Needs’ framework presented in the Animal Welfare Act (2006).

## The problem of synonyms

AZA is the only accreditation body, among those presented in Table 1, that opts to use the word well-being instead of welfare. In their ‘Strategic Framework for the Well-being of Animals’, AZA state that their decision to use the term well-being was deliberate, with well-being seen as a more publicly accessible term that is independent from, but informed by, animal welfare science (AZA 2022). The notion of well-being being easily understood was reflected in research exploring visitor perceptions of animal well-being in North America (Sherman *et al.* 2024), although, in similar work with British and Japanese Zoos, the word welfare was used (Yamanashi *et al.* 2025).

In their welfare strategy, ‘Caring for Wildlife’, WAZA (2015) clearly differentiate between welfare and well-being. In their Glossary of Terms, animal welfare is defined as:

*How an animal copes with the conditions in which it lives. A good state of welfare (as indicated by scientific evidence) results in an animal that is healthy, comfortable, well-nourished, safe, able to express innate behaviour and not suffering from unpleasant states, such as pain and distress* (WAZA 2015; p 82).

Meanwhile, well-being, on the other hand is defined as “*a state of harmony between the animal’s physical and psychological functioning; synonymous with a good welfare state*” (WAZA 2015; p 82). In their 2015 ‘Caring for Wildlife’ Welfare Strategy, WAZA use Maslow’s Hierarchy of Needs to visually depict the difference between welfare and well-being, stating that animals can have good welfare when all basic needs are met, but can only achieve true well-being when all additional needs are fulfilled, representing a higher level of welfare (WAZA 2015).

Other notable work that clearly differentiates between welfare and well-being includes the Subjective Well-being (SWB) Scale, developed by King and Landau (2003), that has been used to assess the overall lived experiences of chimpanzees (*Pan troglodytes*). The SWB scale has also been used with orangutans (*Pongo pygmaeus* and *Pongo abelii)* (Weiss *et al.* 2006), brown capuchins (*Sapajus apella*) (Robinson *et al.* 2016), rhesus macaques (*Macaca mulatta*) (Weiss *et al.* 2011), western lowland gorillas (*Gorilla gorilla gorilla*) (Schaefer & Steklis 2014), and orcas (*Orcinus orca)* (Úbeda *et al.* 2021). Questions in the welfare section of the SWB scale relate to physical health, diet and the environment, among others and those in the subjective well-being section ask raters to assess the amount of time the animal is happy, the degree to which the animal enjoys social interactions and is successful in achieving their goals, and finally, to imagine themselves as the animal and consider how happy they would feel for a week (King & Landau 2003).

Lerner (2008) offers a clear distinction between terms, defining well-being as feelings, pleasure and satisfaction of preferences and welfare as development according to natural selection, coping, fulfilment of needs, natural behaviour, maintained dignity of the animal and a suitable environment and care. Colditz (2023) also clearly differentiates welfare and well-being from one another. Welfare is considered a biological construct, defined by the tripartite model of biological function, natural living and mental state, much like the three orientations model of welfare, first proposed by Fraser and colleagues (1997). Well-being on the other hand, is thought to be more of a philosophical construct and is characterised by eudaimonic, hedonic and social aspects (Colditz 2023).

The connotations of well-being as more subjective, psychological and feelings-based are present across the literature (Whitham & Wielebnowski 2013; Budaev *et al.* 2020; Fletcher *et al.* 2021; Coria-Avila *et al.* 2022; Oldfield & Bonano 2024; Singh *et al.* 2025). It is likened, in some cases, to human well-being, with fulfilment, a sense of purpose, satisfaction of preferences and happiness thought to be central to its definition (Appleby & Sandøe 2002; Düpjan & Dawkins 2022). In their book, *Zoo Animal Welfare*, Rose *et al.* (2025) differentiate welfare and well-being, defining animal welfare as:

*An individual animal’s state as it attempts to cope with the environment that it is currently experiencing. Welfare comprises of behavioural, physical, and psychological components, and will change as an animal experiences different stimuli. In this book, we consider welfare to encompass Broom’s concepts of coping (as an indicator of positive and negative physical, behavioural, and emotional – where measurable – states) alongside Fraser’s ideas on biological functioning, affective states, and enabling natural living.* (Rose *et al.* 2025; p xvii).

Well-being is defined as “*Although often used interchangeably with welfare, this book considers well-being within a human’s perspective of their physical and mental health*” (Rose *et al.* 2025; p xix). However, as Rose *et al.* (2025) themselves state, welfare and well-being are widely reported as being interchangeable words and, as such, meaning the same thing. Table 2 presents an overview of published definitions that conclusively state that welfare and well-being are interchangeable components.

**Table 2.** Published definitions of welfare and well-being interpreted as interchangeable components, including date, exact quote and relevant page number. Table 2. long description.

Author(s)	Date	Quote	Page
Mellor & Reid	1994	“*In their application to non-human animals, ‘welfare’ and ‘well-being’ are interchangeable words*”	3
Hetts *et al.*	2005	“*Welfare often means well-being, happiness, health, prosperity, comfort, or a state of faring well*”	211
Carenzi & Verga	2009	“*Some confusion exists also between the terms welfare and well-being* […] *however the two terms may be probably used as synonyms*”	25
Mason & Veasey	2010	“*Animal welfare* [sometimes termed “well-being”] *is about feelings* […]”	237
Dawkins	2017	“*Animal welfare* [well-being] *can be defined objectively in terms of animal health and what an animal wants*”	2
McMillan & Yeates	2020	“*The extensive overlapping of attributes of the main Well-being concepts of welfare, well-being, QOL* [quality of life], *happiness and SWB [subjective well-being] strongly suggest that several, if not all, of these concepts are the same phenomena.*”	13
Binding *et al.*	2020	“*First, the words ‘welfare’, ‘well being’ or ‘well-being* [hereafter collectively referred to as welfare] […]”	168
Webber *et al.*	2022	“*The concept of animal welfare can be considered equivalent to quality of life and well-being*”	2

Generally, there are more published definitions stating that the two terms, welfare and well-being, are interchangeable components, in comparison to the few reports of the two clearly distinguished. Moreover, within definitions stating that the two terms are in fact, independent of one another, there remain discrepancies regarding how these are differentiated, and as a result, overlap persists leading to increased ambiguity. Conversely, there is clear consistency across definitions whereby welfare and well-being (sometimes alongside additional terms such as quality of life [QoL], subjective well-being [SWB] etc) are remarked upon as being interchangeable synonyms.

## Temporal changes

It appears that there is some weight in the notion of temporal changes that influence the frequency of use of either term. As mentioned, in their ‘Caring for Wildlife’ welfare strategy, WAZA (2015) explicitly differentiate between welfare and well-being. However, in 2020, WAZA published a revised version of their definition of welfare (a shortened version of this was presented in Table 1). This definition also sits within their new welfare strategy, ‘Advancing Animal Welfare’, released in 2025 (WAZA 2025). This second, revised definition, does not offer a clear differentiation between welfare and well-being and, moreover, well-being is not mentioned within the definition nor the strategy itself at all. This shift observed in both the use and understanding of terms from WAZA across a 10-year period further points to temporality in patterns of language use over time.

This is widely acknowledged anecdotally within the zoo sphere but has yet to be formally explored. A brief comparison of usage of terms in peer-reviewed papers was undertaken here to explore this further. Issues in *Zoo Biology* that were published across specified years were reviewed and the number of times that the terms ‘welfare’ and ‘well-being’ were present within each paper were counted. The years 2005, 2015 and 2025 were specifically chosen, linking to the rise in welfare science (early 2000s), the rise in the use of the term well-being and the publication of WAZA’s first animal welfare strategy, ‘Caring for Wildlife’ (mid-2010s; WAZA 2015) and the exponential increase in welfare-related research in zoos, widespread use of the term welfare and the publication of WAZA’s second animal welfare strategy, ‘Advancing Animal Welfare’ (mid-2020s; WAZA 2025). This comparison, however, yielded no results to support the concept of temporality in terms over time, with limited welfare-related research published in the years selected for review. Although the selected years were deliberately chosen, the specific focus here may have restricted the scope of this brief review. While it was not the purpose of this piece to systematically review the change in terminology over time, which instead aimed to offer a high-level overview of the different terms used, future research could explore this concept in more depth by conducting a systematic review of the literature across multiple journals.

## Concluding remarks

This paper offers a synthesis of current applications of the terms welfare and well-being across the zoo sector, highlighting areas of convergence and divergence in welfare conceptualisations. Consistency in the use and understanding of terms is paramount, both within the zoo sector and at a cross-disciplinary level. Definitions of welfare and well-being presented in this paper indicate clear overlap, suggesting that attempts to differentiate the two terms are generally ineffective. Moreover, it has been acknowledged that the use of different synonyms not only leads to increased ambiguity in the definition itself but also leads to grey areas when conducting literature searches, with multiple variations of search terms needed (e.g. well-being, wellbeing, well being, welfare, happiness, wellness, etc) to produce a comprehensive review (McMillan & Yeates 2020; van der Staay *et al.* 2025). It is concluded here that differences in how terms have been defined are attributable to the authors’ discretion in the wording used, possibly influenced by the discipline, location of the author or simply a result of personal preference, rather than a fundamental difference in meaning.

To reduce ambiguity and bring in-line with the scientific discipline of Animal Welfare Science, the authors recommend that the term welfare should be upheld over any other synonyms in future research. To avoid the issue of confusing conceptualisations and synonyms, instead of introducing a new term to reflect positive states (e.g. happiness, well-being etc), it should be emphasised that welfare is a continuum of positive to negative across the whole life of the animal (Dublin Zoo 2025; WAZA 2025).

Rather than adding to the list of definitions of welfare already published, the authors recommend a deviation from a sentence-long definition of welfare and instead encourage future research to regard welfare in terms of its key components. Understanding welfare like this, as a comprehensive and multifaceted concept, allows the flexibility to evolve following advancements in scientific knowledge, acknowledging that the science of animal welfare remains such a rapidly developing field (Green & Mellor 2011; Mellor & Burns 2020). This approach to understanding animal welfare through its key components can be seen in Dublin Zoo’s Animal Welfare Master Plan (Dublin Zoo 2025), WAZA’s welfare strategy, ‘Advancing Animal Welfare’ (WAZA 2025), and in Mellor’s Five Domains Model of Welfare (Mellor *et al.* 2020), whereby similar concepts of: good health, adapting and responding to environmental changes positive, positive interactions with conspecifics and the animal care team, and overall positive lived experience, are emphasised.

This paper offers the first steps in synthesising current applications of the terms welfare and well-being within the zoo sector. As conceptualisations of welfare continue to evolve, clarity in both the terminologies used and their definitions becomes increasingly important. Acknowledgment of how welfare is understood across different accreditation systems can help strengthen communications, consistency, and ultimately improve methods of assessing welfare, continuing to drive the advancement of evidence-based welfare practices.

## Long descriptions

### Table 1. Long description

The table consists of two columns: Accreditation body and Definition.

* A Z A: The state of being comfortable, healthy or happy; achieved by A Z A facilities giving animals lifelong opportunities to thrive.

* B I A Z A: The state of the animal as perceived by the animal itself with regards to its attempts to cope with its environment.

* E A Z A: The physical, behavioural and social well-being of animals through the provision of appropriate conditions for the species involved, including but not necessarily limited to housing, environment, diet, veterinary care and social contact where applicable. The Five Domains Model of Welfare is the primary model accepted by E A Z A for assessing welfare.

* W A Z A: Animal welfare refers to a state that is specific for every individual animal; it is how the animal experiences its own world and life through its association with pleasant experiences specific for that species such as vitality, affection, safety and excitement, or unpleasant experiences such as pain, hunger, fear, boredom, loneliness and frustration.

* Z A A: Positive Welfare/Good Welfare is the welfare state of an animal that is achieved when positive mental experiences are promoted and at the same time negative mental experiences are minimised. Negative Welfare/Poor Welfare is the welfare state of an animal when there are negative mental experiences occurring on a severe, frequent or ongoing basis.

Navigate back to Table 1..

### Table 2. Long description

The table consists of four columns: Author(s), Date, Quote, and Page.

* Mellor and Reid (1994): In their application to non-human animals, ‘welfare’ and ‘well-being’ are interchangeable words. Page 3.

* Hetts et al. (2005): Welfare often means well-being, happiness, health, prosperity, comfort, or a state of faring well. Page 211.

* Carenzi and Verga (2009): Some confusion exists also between the terms welfare and well-being however the two terms may be probably used as synonyms. Page 25.

* Mason and Veasey (2010): Animal welfare [sometimes termed ‘well-being’] is about feelings. Page 237.

* Dawkins (2017): Animal welfare [well-being] can be defined objectively in terms of animal health and what an animal wants. Page 2.

* McMillan and Yeates (2020): The extensive overlapping of attributes of the main Well-being concepts of welfare, well-being, Q O L [quality of life], happiness and S W B [subjective well-being] strongly suggest that several, if not all, of these concepts are the same phenomena. Page 13.

* Binding et al. (2020): First, the words ‘welfare’, ‘well being’ or ‘well-being’ [hereafter collectively referred to as welfare]. Page 168.

* Webber et al. (2022): The concept of animal welfare can be considered equivalent to quality of life and well-being. Page 2.

Navigate back to Table 2..
